# Targeted Next Generation Sequencing and Diagnosis of Congenital Hemolytic Anemias: A Three Years Experience Monocentric Study

**DOI:** 10.3389/fphys.2021.684569

**Published:** 2021-05-21

**Authors:** Elisa Fermo, Cristina Vercellati, Anna Paola Marcello, Ebru Yilmaz Keskin, Silverio Perrotta, Anna Zaninoni, Valentina Brancaleoni, Alberto Zanella, Juri A. Giannotta, Wilma Barcellini, Paola Bianchi

**Affiliations:** ^1^UOS Fisiopatologia delle Anemie, UOC Ematologia, Fondazione IRCCS Ca’ Granda Ospedale Maggiore Policlinico, Milan, Italy; ^2^Department of Pediatric Hematology and Oncology, Suleyman Demirel University, Isparta, Turkey; ^3^Dipartimento della Donna, del Bambino e di Chirurgia Generale e Specialistica, Università degli Studi della Campania “Luigi Vanvitelli,” Naples, Italy; ^4^UOC Medicina Generale, Fondazione IRCCS Ca’ Granda Ospedale Maggiore Policlinico, Milan, Italy

**Keywords:** congenital hemolytic anemia, targeted-NGS, pathogenic variants, red blood cells, differential diagnosis

## Abstract

Congenital hemolytic anemias (CHAs) are heterogeneous and rare disorders caused by alterations in structure, membrane transport, metabolism, or red blood cell production. The pathophysiology of these diseases, in particular the rarest, is often poorly understood, and easy-to-apply tools for diagnosis, clinical management, and patient stratification are still lacking. We report the 3-years monocentric experience with a 43 genes targeted Next Generation Sequencing (t-NGS) panel in diagnosis of CHAs; 122 patients from 105 unrelated families were investigated and the results compared with conventional laboratory pathway. Patients were divided in two groups: 1) cases diagnosed with hematologic investigations to be confirmed at molecular level, and 2) patients with unexplained anemia after extensive hematologic investigation. The overall sensitivity of t-NGS was 74 and 35% for families of groups 1 and 2, respectively. Inside this cohort of patients we identified 26 new pathogenic variants confirmed by functional evidence. The implementation of laboratory work-up with t-NGS increased the number of diagnoses in cases with unexplained anemia; cytoskeleton defects are well detected by conventional tools, deserving t-NGS to atypical cases; the diagnosis of Gardos channelopathy, some enzyme deficiencies, familial siterosterolemia, X-linked defects in females and other rare and ultra-rare diseases definitely benefits of t-NGS approaches.

## Introduction

Congenital hemolytic anemias (CHAs) comprise a group of very heterogeneous and rare disorders caused by alterations in structure, transport functions, metabolism, or defective production of red blood cells (RBCs). Since the pathophysiology of some rare forms is poorly understood, these disorders represent a group of diseases that still lack easy-to-apply tools for diagnosis, clinical management, and patient stratification.

The laboratory diagnostic pathway of CHAs was historically based on sequential steps using a panel of functional analyses that investigate the RBC membrane and metabolism. The first diagnostic step relies on hematological tests (complete blood count, red cell indices, hemolysis markers), peripheral blood smear examination, osmotic fragility tests and eosin-5-maleimide (EMA) binding test. The second includes biochemical tests such as quantitative assay of RBC enzymes activity, sodium dodecyl sulphate-polyacrylamide gel electrophoresis (SDS-PAGE) for the diagnosis of membranopathies, and specialized investigations requiring instruments which are available only in reference Centers, as ektacytometry (King et al., 2015; Fermo et al., 2021). The molecular characterization of the affected genes by traditional Sanger sequencing was usually, up to some years ago, the last step allowing the definitive confirmation of the diagnosis. Despite this, even after extensive and complete investigation, the differential diagnosis of these disorders may be difficult, and some patients remain undiagnosed with a consequent negative impact on clinical follow up, risk of inappropriate therapeutic decisions and lack of access to new specific treatments.

The advent and recent progresses on next generation sequencing (NGS) technologies has radically changed the diagnostic approach to CHAs, often placing the genetic analysis as a first line screening tool; different NGS strategies have been developed in the last years including targeted panels and whole exome sequencing (WES), with a progressive reduction of costs that allowed their routinely use (Steinberg-Shemer and Tamary, 2020; Russo et al., 2020). Targeted-NGS panels have been developed and applied by several groups, being currently the preferred approach for the molecular diagnosis of CHAs; custom panels include different numbers of genes and have been reported to have a wide range of diagnostic efficacy (38–90%) depending on the number of genes included and on the characteristics of the patients studied (Agarwal et al., 2016; Del Orbe Barreto et al., 2016; Niss et al., 2016; Roy et al., 2016; Russo et al., 2018; Shefer Averbuch et al., 2018; Choi et al., 2019; Kedar et al., 2019a,b; Svidnicki et al., 2020). In this paper we report the 3 years monocentric experience in targeted-NGS and diagnosis of CHAs, and we compare the efficacy of this methodological approach with the conventional laboratory diagnosis.

## Materials and Methods

### Patients

Among the patients referred to our Institut (Pathophysiology of Anemias Unit, Fondazione IRCCS Ca’ Granda Ospedale Maggiore of Milan–Italy) between 2017 and 2019 with a clinical suspect of CHAs we examined a cohort of 122 patients from 105 unrelated families (51% males and 49% females, median age 27 years, range 0–73 years). The inclusion criteria in the study were: a diagnostic indication for congenital hemoltic anemia; the specific request from clinicians to perform/complete diagnosis at molecular level; a specific scientific interest in cases with intra-family variability or atypical presentation; all cases with unexplained phenotype despite extensive investigations; signed informed consent to perform molecular investigations. Samples were collected from patients and controls during diagnostic procedures after obtaining informed consent and approval from the Institutional Human Research Committee. For patients under the age of 18, written informed consent was obtained from the parents. The procedures followed were in accordance with the Helsinki International ethical standards on human experimentation.

All patients but 18 (received after shipping from abroad) underwent extensive hematologic investigations, including: RBC morphology, complete blood count, hemolysis markers, screening for abnormal/unstable hemoglobins, direct antiglobulin test, osmotic fragility tests, EMA binding test (Bianchi et al., 2012), and RBC enzymes activities determination (hexokinase, glucosephosphate isomerase, phosphofructokinase, glyceraldehyde-P-dehydrogenase, phos phoglycerate kinase, pyruvate kinase, glucose-6-phosphate dehydrogenase, 6-phosphogluconate dehydrogenase, adenylate kinase) (Beutler et al., 1977).

In the vast majority of cases we also performed Osmoscan analysis by LoRRca MaxSis (Laser-Assisted Optical Rotational Cell Analyzer, Mechatronics, NL) (Zaninoni et al., 2018, see Supplementary Materials for more details), and RBC membrane protein analysis by SDS–PAGE performed according to Laemmli (1970) and Fairbanks et al. (1971) with minor modifications (Mariani et al., 2008).

In the remaining 18 cases (15 families), when possible, a minimum panel of investigation was performed (i.e., EMA binding test, RBC enzyme assay, Osmoscan) and evaluated together with clinical and laboratory information received from the local centers.

In 60 patients from 51 families a definite diagnostic orientation was obtained after first and second level hematological investigations. In particular, 6 were suspected of RBC enzyme defects, 28 of structural membrane defects (hereditary spherocytosis, elliptocytosis, pyropoikilocytosis, HS/HE/HPP), 10 of stomatocytosis and 7 were classified as congenital dyserythropoietic anemia.

In the remaining 62 cases (from 54 families) the laboratory investigations did not allow a clear diagnosis.

### NGS Sequencing and Analysis

Genomic DNA was extracted from peripheral blood, using standard manual methods and quantified by Nandrop One (Thermo Scientific, Italy). When available, relatives of affected cases were also enrolled to analyze allelic segregation and correctly assess the pathogenicity of each variant.

Using SureDesign software (Agilent) we created a NGS based panel containing 43 genes already described as disease causing for RBC membrane disorders (*n* = 14 genes), enzymopathies (*n* = 18), dyserythropoietic and sideroblastic anemias (*n* = 11) (Supplementary Table 1). For the probe design, coding regions, 5’UTR, 3’UTR, 50 bp flanking splice junctions were selected as regions of interest. Sequence length was set at 150 × 2 nucleotides and the average target coverage was 99.52%. The panel was validated on previously characterized patients affected by CHAs.

Libraries were obtained by HaloPlexHS Target Enrichment System Kit (Agilent) following the instruction’s manufacturer. Samples were pooled (average 22 samples) and loaded at 7 pM on MiSeq platform using a v2-300 cycle reagent kit (Illumina). Sequencing reads were aligned against reference genome (UCSC hg19) and variants were called and annotated using the SureCall software (Agilent Technologies).

Filter settings were as follows: variant call quality threshold > 100; minimum number of reads supporting variant allele > 10; mutant allele frequency ≥ 25%; minor allele frequency (MAF) < 1% of the population. Common pathogenic modifiers of hereditary spherocytosis known to be present above the 1% cutoff, e.g., *SPTA1* LELY (Low Expression Lyon) allele (p.L1858V, found in association with the intronic variant c.6531-12 c > t), or the SPTA-Bughill allele (p.A970D) usually found in linkage with the LEPRA (Low Expression PRAgue) allele (SPTA c.4339-99 c > t) were also included.

Targeted filtering and annotation of protein-changing variants were performed using the wANNOVAR web tool^1^.

Variants were assessed by mutation prediction and conservation programs including SIFT^2^, polyphen-2 (Polymorphism Phenotyping v2)^3^ and MutationTaster^4^; pathogenicity was evaluated according to the guidelines of American College of Medical Genetics and Genomics (ACMG) (Richards et al., 2015) using the online tool VarSome^5^ (Kopanos et al., 2019).

Variants previously classified as pathogenic by databases such as ClinVar, HGMD, dbSNP, deleterious variants expected to produce truncated or abnormal protein, or splice site variants were considered as causatives. Variants of unknown significance (VUSs) were reported only if found in genes relevant to the primary indication for testing.

The mutations identified were confirmed by Sanger method (ABI PRISM 310 Genetic Analyzer, Applied Biosystems, Warrington, United Kingdom) using the Big Dye Terminator Cycle Sequencing Kit (Applied Biosystems, Warrington, United Kingdom).

Nucleotide numbering reflects cDNA numbering with + 1 corresponding to the A of ATG translation initiation codon in the reference sequence, according to the nomenclature for the description of sequence variants of Human Genome Variation Society^6^. The initiation codon is codon 1.

According with ACMG guidelines, well-established *in vitro* functional studies were performed to assess pathogenicity of VUSs, in particular: spectrophotometric enzyme assay in the suspect of RBC enzyme defects, SDS-PAGE electrophoresis analysis of RBC membrane proteins in the suspect of membrane defects and CDAII, ektacytometry and patch-clamp analysis in case of novel missense variants in *PIEZO1* and *KCNN4* mutations.

## Results

The 122 patients examined have been divided in two groups: (1) patients who reached a diagnosis after first and second level hematologic investigations, to be confirmed at molecular level; (2) patients with unexplained chronic hemolytic anemia after extensive hematologic investigations.

Patients belonging to group 1 (60 patients, 51 families) were selected among the entire cohort of patients with CHAs studied between 2017 and 2019 basing on complexity of clinical presentation, intra-family clinical variability or incomplete molecular characterization by Sanger (e.g., enzyme defects or CDA cases). All the available patients who didn’t reach a definitive diagnosis in the same period were investigated and included in group 2 (62 cases from 54 families, 39 of them studied in our Center, the other 15 received from abroad).

### Group 1

**FIGURE 1:**
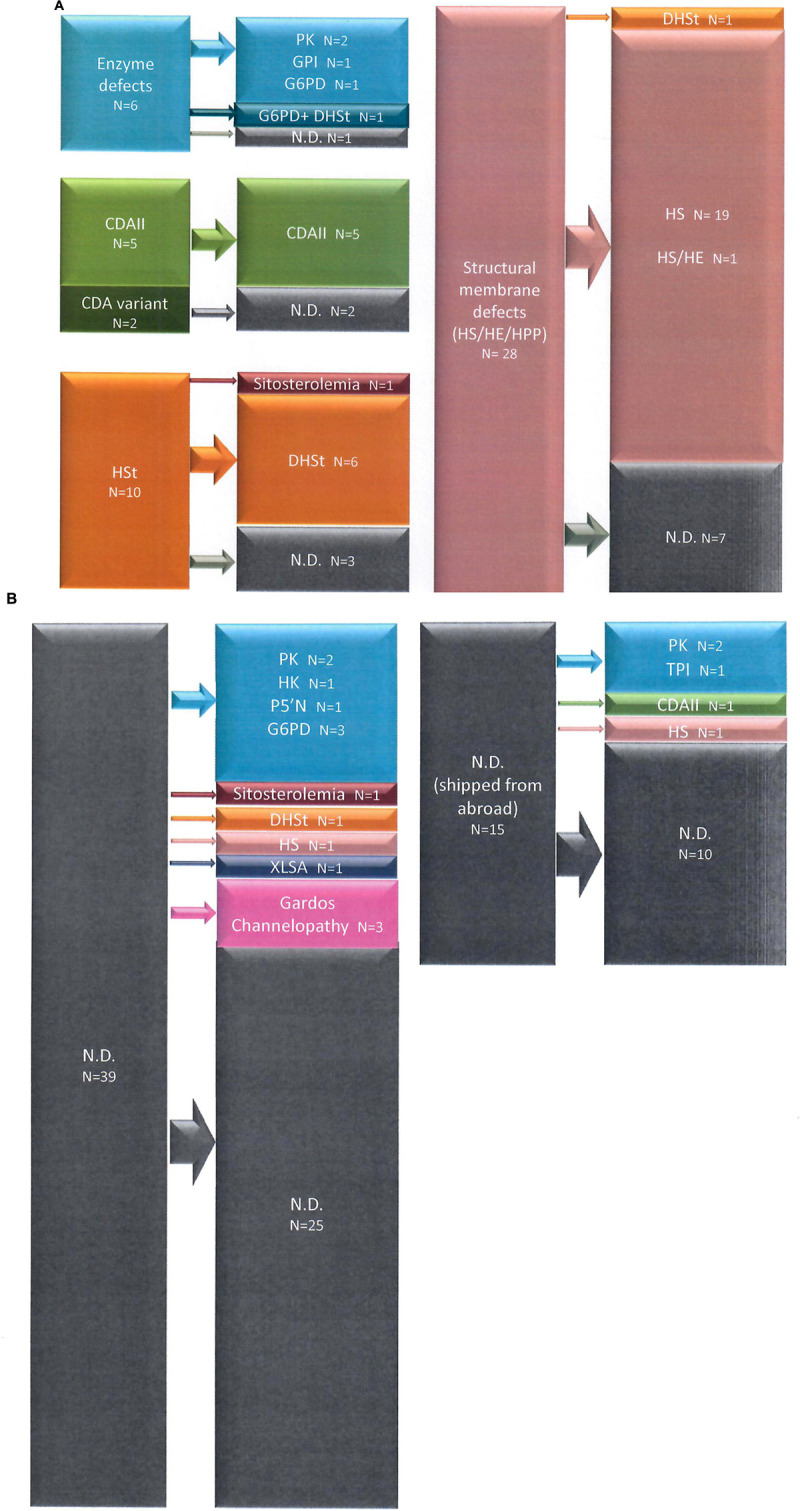
Diagnostic suspect after first and second level laboratory investigation and final diagnosis after t-NGS in Group 1 **(A)** and Group 2 **(B)** patients.

We were able to identify pathogenic variants in 38/51 families belonging to Group 1, with an overall sensitivity of the t-NGS of 74% (Figure 1A and Table 1).

**TABLE 1:** Results of NGS analysis in 64 patients with congenital hemolytic anemia.

Pt. ID	Laboratory diagnosis	Gene	Transcript	Mutation	Effect	Status	rs	Pathogenicity	Final diagnosis	Additional findings
**Group 1**										
1-1	Enzyme defect	*PKLR*	NM_000298.5	c.1618 + 2t>c	Abn. splicing	Comp het	rs983394596	5-P	PK deficiency	
		*PKLR*	NM_000298.5	c.1456C>T	p.R486W	Comp het	rs116100695	5-P		
2-1	Enzyme defect	*PKLR*	NM_000298.5	c.-73G>C	Promoter variant	Comp het		5-P	PK deficiency	*SPTA1* a - lely
		*PKLR*	NM_000298.5	c.1456C>T	p.R486W	Comp het	rs116100695	5-P		
3-1	Enzyme defect	*GPI*	NM_000175.3	c.1415G>A	p.R472H	Het	rs148811525	5-P	GPI deficiency	
4-1	Enzyme defect	*G6PD*	NM_001042351.1	c.202G>A	p.V68M	Hem	rs1050828	5-P	G6PD deficiency	*SPTA1* a - lely
5-1	Enzyme defect	*G6PD*	NM_001042351.1	c.563C>T	p.S188F	Hom	rs5030868	5-P	G6PD def./DHSt	
		*PIEZO1*	NM_001142864.3	c.7367G>A	p.R2456H	Het	rs587776988	5-P		
5-2	Enzyme defect	*G6PD*	NM_001042351.1	c.563C>T	p.S188F	Hom	rs5030868	5-P	G6PD def./DHSt	
		*PIEZO1*	NM_001142864.3	c.7367G>A	p.R2456H	Het	rs587776988	5-P		
6-1	CDAII	*SEC23B*	NM_006363.4	c.40C>T	p.R14W	Comp het	rs121918222	5-P	CDAII	*SPTA1* a - lely
		*SEC23B*	NM_006363.4	c.689 + 1 g>a	Abn. splicing	Comp het	rs398124226	5-P		
7-1	CDAII	*SEC23B*	NM_006363.4	c.325G>A	p.E109K	Comp het	rs121918221	5-P	CDAII	
		*SEC23B*	NM_006363.4	c.2270A>C	p.H757P	Comp het	rs1331894607	5-P		
8-1	CDAII	*SEC23B*	NM_006363.4	c.40C>T	p.R14W	Comp het	rs121918222	5-P	CDAII	
		*SEC23B*	NM_006363.4	c.1821delT	p.H608IfsX7	Comp het		5-P		
9-1	CDAII	*SEC23B*	NM_006363.4	c.235C>T	p.R79X	Het	rs150263014	5-P	CDAII	
10-1	CDAII	*SEC23B*	NM_006363.4	c.40C>T	p.R14W	Het	rs121918222	5-P	CDAII	*SPTA1* a - lely
11-1	HSt	*ABCG8*	NM_022437.2	c.788G>A	p.R263Q	Hom	rs137852990	5-P	Sitosterolemia	
12-1	HSt	*PIEZO1*	NM_001142864.3	c.6574C>A	p.L2192I	Het		4-LP	DHSt	
12-2	HSt	*PIEZO1*	NM_001142864.3	c.6574C>A	p.L2192I	Het		4-LP	DHSt	
13-1	HSt	*PIEZO1*	NM_001142864.3	c.7367G>A	p.R2456H	Het	rs587776988	5-P	DHSt	
14-1	HSt	*PIEZO1*	NM_001142864.3	c.6007G>A	p.A2003T	Het		4-LP	DHSt	
15-1	HSt	*PIEZO1*	NM_001142864.3	c.6328C>T	p.R2110W	Het	rs776531529	5-P	DHSt	*SPTA1* a - lely
16-1	HSt	*PIEZO1*	NM_001142864.3	c.1815G>A	p.M605I	Het		4-LP	DHSt	
16-2	HSt	*PIEZO1*	NM_001142864.3	c.1815G>A	p.M605I	Het		4-LP	DHSt	
17-1	HSt	*PIEZO1*	NM_001142864.3	c.1815G>A	p.M605I	Het		4-LP	DHSt	*SPTA1* a - lely
18-1	Membrane defect	*PIEZO1*	NM_001142864.3	c.1792G>A	p.V598M	Het		4-LP	DHSt	
19-1	Membrane defect	*SLC4A1*	NM_000342.3	c.1469G>A	p.R490H	Het		5-P	HS	
19-2	Membrane defect	*SLC4A1*	NM_000342.3	c.1469G>A	p.R490H	Comp het		5-P	HS	*SPTA1* a - lely
		***SLC4A1***	**NM_000342.3**	**c.558_572del15bp**	**p.QPLLPQ186Q**	Comp het		4-LP		
		***SLC4A1***	**NM_000342.3**	**c.1627-1g>t**	**Abn. splicing**	Comp het		5-P		
20-1	Membrane defect	***SPTA1***	**NM_003126.3**	**c.3477 + 1g>c**	**Abn. splicing**	Comp het		5-P	HS	*SPTA1* a - lely
		*SPTA1*	NM_003126.3	c.3139C>T	p.R1047X	Comp het		5-P		
21-1	Membrane defect	***SPTB***	**NM_001355436.1**	**c.154C>T**	**p.R52W**	Het		4-LP	HS	*SPTA1* a - lely
22-1	Membrane defect	***SPTB***	**NM_001355436.1**	**c.3106_3107insC**	p.Q1036Pfs*37	Het		5-P	HS	*SPTA1* a - lely
23-1	Membrane defect	***SPTB***	**NM_001355436.1**	**c.3936G>A**	**p.W1312X**	Het		5-P	HS	*PKLR* p.R486W
24-1	Membrane defect	*SLC4A1*	NM_000342.3	c.1462G>A	p.V488M	Het	rs28931584	5-P	HS	
25-1	Membrane defect	***SLC4A1***	**NM_000342.3**	**c.2609G>A**	**p.R870Q**	Het	rs746426065	4-LP	HS	*SPTA1* a - lely
26-1	Membrane defect	*SLC4A1*	NM_000342.3	c.2279G>A	p.R760Q	Het	rs121912755	5-P	HS	
27-1	Membrane defect	***SLC4A1***	**NM_000342.3**	**c.2510C>A**	**p.T837K**	Het		4-LP	HS	
28-1	Membrane defect	***SLC4A1***	**NM_000342.3**	**c.2269A>T**	**p.K757X**	Het		5-P	HS	*SEC23B* c.689 + 1G>A *SPTA1* a - lely
29-1	Membrane defect	***ANK1***	**NM_000037.3**	**c.4541delA**	**p.Y1514SfsX33**	Het		5-P	HS	*SPTA1* a - lely
30-1	Membrane defect	***ANK1***	**NM_000037.3**	**c.1430_1431insGTGC**	**p.A478CfsX17**	Het		5-P	HS	
31-1	Membrane defect	***ANK1***	**NM_000037.3**	**c.4057C>T**	**p.Q1353X**	Het		5-P	HS	*SPTA1* a - lely
32-1	Membrane defect	***SPTB***	**NM_001355436.1**	**c.4842 + 1g>c**	**Abn. splicing**	Het		5-P	HS	*SPTA1* a - lely
33-1	Membrane defect	***EPB42***	**NM_000119.2**	**c.922G>C**	**p.V308L/Abn. splicing**	Comp het	rs772330879	4-LP	HS	
		***EPB42***	**NM_000119.2**	**c.413C>T**	**p.T138I**	Comp het		4-LP		
34-1	Membrane defect	***SLC4A1***	**NM_000342.3**	**c.2312G>T**	**p.G771V/Abn. splicing**	Het		4-LP	HS	
35-1	Membrane defect	*SLC4A1*	NM_000342.3	c.163delC	p.H55TfsX11	Het		5-P	HS	
36-1	Membrane defect	***SPTB***	**NM_001355436.1**	**c.1024C>T**	**p.Q342X**	Het		5-P	HS	
37-1	Membrane defect	*SLC4A1*	NM_000342.3	c.2423G>A	p.R808H	Het	rs866727908	4-LP	HS	
38-1	Membrane defect	*SPTA1*	NM_003126.3	c.460_462dupTTG	p.L155dup	Het	rs757679761	4-LP	HE	
38-2	Membrane defect	*SPTA1*	NM_003126.3	c.460_462dupTTG	p.L155dup	Het	rs757679761	4-LP	HS/HE	
		***SLC4A1***	**NM_000342.3**	**c.1530C>G**	**p.S510R**	Het		3-VUS		
**Group 2**										
39-1	n.d	*PKLR*	NM_000298.5	c.1456C>T	p.R486W	Comp het	rs116100695	5-P	PK deficiency	
		*PKLR*	NM_000298.5	c.1151C>T	p.T384M	Comp het	rs74315362	5-P		
40-1	n.d	*PKLR*	NM_000298.5	c.1456C>T	p.R486W	Comp het	rs116100695	5-P	PK deficiency	*SPTA1* a - lely
		*PKLR*	NM_000298.5	c.958G>A	p.V320M	Comp het		5-P		
41-1	n.d	***HK1***	**NM_033496.2**	**c.34C>T**	**p.R12X**	Comp het	rs756166032	5-P	HK deficiency	
		***HK1***	**NM_033496.2**	**c.1351G>C**	**p.G451R**	Comp het		4-LP		
42-1	n.d	***NT5C3A***	**NM_016489.12**	**c.64C>T**	**p.R22X**	Hom	rs753346459	5-P	P5’N deficiency	*SPTA1* a - lely
43-1	n.d	*G6PD*	NM_001042351.1	c.563C>T	p.S188F	Het	rs5030868	5-P	G6PD deficiency	
44-1	n.d	*G6PD*	NM_001042351.1	c.1160G>A	p.R387H	Hem	rs137852321	5-P	G6PD deficiency	
45-1	n.d	*G6PD*	NM_001042351.1	c.1180G>C	p.V394L	Hem	rs137852335	5-P	G6PD deficiency	*NT5C3A* p.N178H
46-1	n.d	***ABCG5***	**NM_022436.2**	**c.1374C>G**	**p.Y458X**	Hom		5-P	Sitosterolemia	
47-1	n.d	*PIEZO1*	NM_001142864.3	c.7367G>A	p.R2456H	Het	rs587776988	5-P	DHSt	
47-2	n.d	*PIEZO1*	NM_001142864.3	c.7367G>A	p.R2456H	Het	rs587776988	5-P	DHSt	
48-1	n.d	***SPTB***	**NM_001355436.1**	**c.2278C>T**	**p.Q760X**	Het		5-P	HS	*SPTA1* a - lely
49-1	n.d	***ALAS2***	**NM_000032.5**	**c.1571A>G**	**p.H524R**	Het		4-LP	XLSA	*SPTA1* a - lely
50-1	n.d	*KCNN4*	NM_002250.2	c.1055G>A	p.R352H	Het	rs774455945	5-P	Gardos channelopathy	*SPTA1* a - lely
51-1	n.d	*KCNN4*	NM_002250.2	c.940T>C	p.S314P	Het		4-LP	Gardos channelopathy	*AK1* p.G50N
52-1	n.d	*KCNN4*	NM_002250.2	c.1055G>A	p.R352H	Het	rs774455945	5-P	Gardos channelopathy	*SPTA1* a - lely
53-1	n.d	*PKLR*	NM_000298.5	c.581G>C	p.R194P	Hom		4-LP	PK deficiency	
54-1	n.d	***PKLR***	**NM_000298.5**	**c.1591C>A**	**p.R531S**	Hom		4-LP	PK deficiency	
55-1	n.d	*TPI1*	NM_000365.5	c.315G>T	p.E105D	Hom	rs121964845	5-P	TPI deficiency	
56-1	n.d	*SEC23B*	NM_006363.4	c.40C>T	p.R14W	Comp het	rs121918222	5-P	CDAII	
		*SEC23B*	NM_006363.4	c.490delG	p.V164Wfs*3	Comp het	rs776983439	5-P		
57-1	n.d	***SPTB***	**NM_001355436.1**	**c.2647delC**	p.L883Wfs*15	Het		5-P	HS	*SPTA1* a - lely
57-2	n.d	***SPTB***	**NM_001355436.1**	**c.2647delC**	p.L883Wfs*15	Het		5-P	HS	

*Newly detected variants are reported in bold. Het, heterozygous; Comp het, compound heterozygous; Hom, homozygous; Hem, hemizygous; 5-P, pathogenic; 4-LP, likely pathogenic; 3-VUS, variant of uncertain significance; n.d, not determined. The ClinVar accessions for the new variants here reported are SCV001469066-SCV001469086.*

#### Enzyme Defects

Eight cases from six families had a biochemical diagnosis of enzyme defect; two were confirmed to have pyruvate kinase (PK) deficiency, three from two families glucose-6-phosphate dehydrogenase (G6PD) deficiency and one was found to have one mutation in *GPI* gene, in compound heterozygosity with the polymorphism c.489A > G (p.Gly163 = rs1801015), possibly associated to splicing alterations (Fermo et al., 2019); in two related cases no mutations in *PKLR* gene were found in spite of a slightly decreased PK activity detected by enzymatic assay.

Among these cases with enzyme defects, in four patients from three families a coinheritance of hereditary stomatocytosis was also suspected due to the presence of stomatocytes at peripheral blood smear and left-shifted Osmoscan curve; only in two of them (5-1 and 5-2) in addition to G6PD deficiency we found a mutation in *PIEZO1* gene causing hereditary stomatocytosis.

#### Congenital Dyserythropoietic Anaemias (CDAs)

Seven cases had a suspect of congenital dyserythropoietic anemia; five were classified as CDAII on the basis of band 3 deglycosylation at SDS-PAGE analysis, and two had an atypical CDA variant with distinct morphological abnormalities at bone marrow examination. Three out of five CDAII cases displayed biallelic mutations in *SEC23B* gene, whereas in two cases (9-1 and 10-1) we were able to find only one pathogenic variant; in the two cases with the atypical CDA we didn’t find any pathological mutation in the genes associated to CDAs included in our panel.

#### Hereditary Stomatocytosis

Thirteen patients from 10 families had a suspect of hereditary stomatocytosis based on presence of stomatocytes at peripheral blood smear and/or abnormalities at ektacytometric curve; eight of them (from six families) were confirmed to have dehydrated stomatocytosis caused by *PIEZO1* gene pathogenic variants. In one case previously diagnosed as overhydrated stomatocytosis (11-1) based on a clear right-shifted ektacytometric curve, we found mutations in *ABCG8* gene responsible for sitosterolemia. In the remaining case no pathogenic variants in genes associated with hereditary stomatocytosis were identified.

#### Membrane Defects

**FIGURE 2:**
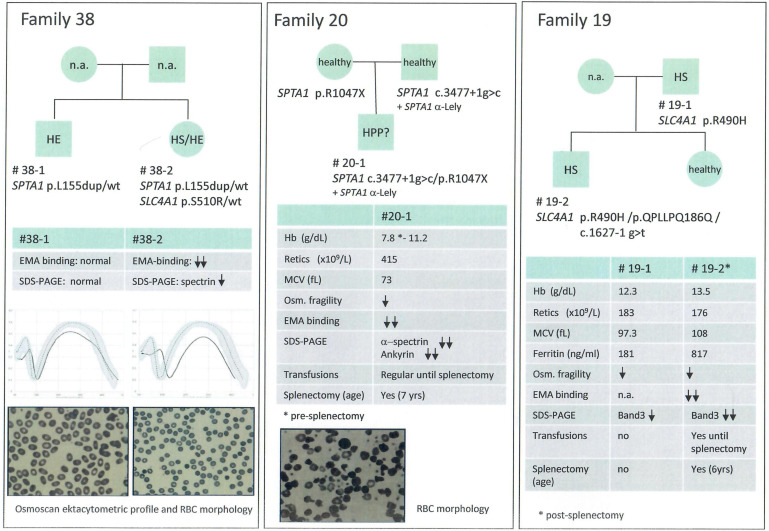
Families with erythrocyte membrane defects and complex phenoptypes and/or intrafamily variability.

We included in this group chronic hemolytic anemias due to altered RBC membrane structural organization, as HS, HE, HPP; 32 cases from 28 families have received such diagnosis based on first and second level laboratory investigations. In 23 cases from 21 families we found pathogenic variants. One patient with a previous diagnosis of HS was found to be affected by dehydrated stomatocytosis (DHSt); in the remaining cases, mutations in genes responsible for HS and HE were identified. In particular among them we studied 3 families with a complex phenotype: case 38-2 (mixed HS/HE phenotype) carried two variants in *SPTA1* and *SLC4A1*, whereas the brother with typical HE (38-1) had only the *SPTA1* variant. Case 20-1, presenting severe HPP with combined 68% spectrin and 56% ankyrin deficiency, had two different mutations in *SPTA1* gene transmitted by the parents, one of them associated in *cis* with the alpha-LELY allele. Finally, case 19-2 (severe HS with 50% band3 deficiency) had 3 different variants in *SLC4A1* gene, the father (19-1) presenting only one missense mutation, thus justifying intra-family clinical variability (Figure 2).

### Group 2

Inside this group we were able to identify pathogenic variants in 19/54 families (21/62 patients), with an overall sensitivity of the t-NGS of 35% (Figure 1B and Table 1).

Ten families were found to have pathogenic variants in RBC enzymes. In five of them (2 PK, 2 G6PD and 1 hexokinase (HK) deficiency, cases 39-1, 40-1, 43-1, 44-1, and 41-1) the enzyme activity was previously tested and found to be normal; in other four cases enzyme activity was not tested for sample unavailability. One case had known G6PD deficiency that was initially not considered during the laboratory screening based on chronic hemolysis. Triosephosphate isomerase (TPI) deficiency was diagnosed in a 7 months old child presenting at the time of the study only hemolytic anemia without neuromuscular manifestations.

Three unrelated cases were found to have pathogenic variants in *KCNN4* gene associated with Gardos channelopathy, two of them carrying the common variant p.R352H and one a private variant p.S314P resulting in channel activation (Fermo et al., 2020).

Only one family with two affected siblings (47-1 and 47-2), addressed to our attention in the suspect of PK deficiency in absence of pathogenic variants in *PKLR* gene, carried a PIEZO1 pathogenic variant. Patient 46-1, who had 17% of stomatocytes at peripheral blood smear without any abnormality of the Osmoscan curve, was diagnosed with sitosterolemia due to *ABCG5* mutation p.Y458X. Finally, NGS analysis allowed the diagnosis of X-linked sideroblastic anemia (XLSA) in a woman affected by unexplained macrocytic anemia since birth (case 49-1, *ALAS2* mutation p.H524R). Human androgen receptor gene (HUMARA) analysis showed skewed X-Chromosome inactivation pattern; the mutation was transmitted by the asymptomatic mother.

### New Variants Identified

Inside the analyzed cohort we identified 26 new variants, 20 of them associated with structural membrane defects (*SLC4A1*: p.QPLLPQ186Q, p.R870Q, p.T837K, p.K757X, p.G771V, p.S510R, c.1627-1g>t; *SPTB*: p.R52W, p.Q1036PfsX37, p.W1312X, p.Q342X, p.Q760X, p.L883WfsX15, c.4842+1g>c; *ANK1*: p.Y1514SfsX33, p.A478CfsX17, p.Q1353X; *SPTA1*: c.3477+1g>c; *EPB42* p.V308L, p.T138I). The remaining new variants were identified in enzyme genes (*PKLR*: p.R531S; *HK1*: p.R12X, p.G451R; *NT5C3A*: p.R22X), and in genes associated with sitosterolemia (*ABCG5*: p.Y458X) and sideroblastic anemia (*ALAS2*: p.H524R). All new variants were classified as pathogenic or likely pathogenic by *in silico* analysis, according with transmission, clinical phenotype and functional laboratory evidences. Only one VUS variant in *SLC4A1* gene was included and considered as pathogenic because clearly fitting with patients phenotype in a family with a suspected combined transmission of HS and HE (case 38-2) (Figure 2).

## Discussion

The observed sensitivity of NGS in the two groups of patients analyzed is very different, varying from 74% (patients with a specific diagnostic orientation, Group 1) to 35% (hemolytic patients with no diagnosis after laboratory investigations, or not investigated due long shipping conditions, Group 2).

These data are in line with the available literature (Russo et al., 2018; Bianchi et al., 2020; Steinberg-Shemer and Tamary, 2020) and may explain the heterogeneous sensitivity that, independently from the number of genes analyzed, is often reported for these techniques. For example, the overall sensitivity was around 38.6% in a large series of 57 hemolytic patients with no definitive diagnosis (Roy et al., 2016) and 45.8% in a group of undiagnosed hemolytic patients studied with a combination of two-step t-NGS platforms covering about 100 genes (Russo et al., 2018). Different results are found when NGS is performed on well characterized patient subsets, for example in series of HS patients, where the reported sensitivity increased to 70–100% (Chonat et al., 2019; van Vuren et al., 2019; Xue et al., 2019; Vives-Corrons et al., 2021).

In this view, a sensitivity of 75%, as obtained in our Group 1, may be expected for t-NGS, considering some technical limitations of this technique (loss of variant calling, presence of pathogenic variants in not sequenced regions, copy number variations not always revealed), or to the presence of diseases-causing variants in new genes or genes not included in the panels. In such cases, results could be further improved by considering other technical approaches (WES or WGS) (Mansour-Hendili et al., 2020).

In a portion of cases reported in literature up to 45%, the genetic testing changed the initial diagnosis, in particular when transfusion dependence prevented laboratory investigations from being carried out, and the suspect was based only on clinical features (Russo et al., 2018). In our experience, when detailed clinical history is available together with fresh blood samples collected far from transfusions, first and second level laboratory diagnosis for CHAs has a good level of specificity, as demonstrated by the finding of only two patients who changed diagnosis after NGS analysis inside group 1: one patient previously diagnosed as HS had indeed a pathogenic variant in *PIEZO1* gene, and one case with a long-lasting diagnosis of overhydrated stomatocytosis (done on morphological bases, with 18% of stomatocytes and right shift of ektacytometry curve) was indeed a case of familial sitosterolemia with homozygous mutation p.R263Q in *ABCG8* gene (Berge et al., 2000). In both cases the molecular diagnosis changed the clinical management: in the first case the patient was warned about the thrombotic risk associated with splenectomy in hereditary stomatocytosis, and in the second case suggestions were given about dietary reduction of plant sterols consumption, to reduce cardiovascular risk that is associated with familial sitosterolemia (Tzavella et al., 2017). Patients affected by hereditary sitosterolemia may display an extremely variable clinical phenotype, in some cases restricted to abnormal hematological features, leading to misdiagnosis with hereditary stomatocytosis, immune thrombocytopenia (ITP), or Evans syndrome. The mechanisms of hematologic changes in these patients are so far unexplained, and a toxic effect of plant sterols on red blood cells has been hypothezied from *in vitro* experiments (Wang et al., 2012). The finding of two cases in our series, in line with increasing reports of the last years (Wang et al., 2014; Zheng et al., 2019), suggest that sitosterolemia may have an underestimated prevalence, and it should be taken in account in differential diagnosis of CHAs, in particular when stomatocytosis and concomitant macrothrombocytopenia are observed.

Combined defects of red cell membrane and/or metabolism are very rare, and the fact that carriership for a metabolic defect might modify the clinical expression of a membrane defect is still debated (van Zwieten et al., 2015; Fermo et al., 2017). However, the detection of cases with concomitant red cell defects has increased in the last years after the introduction of NGS analysis (Russo et al., 2018; Mansour-Hendili et al., 2020) suggesting that these conditions might not be so rare, also possibly due to protection toward malaria infection. In this series we found the association of G6PD deficiency and *PIEZO1* stomatocytosis in two female patients (mother and daughter) with suspected hereditary stomatocytosis. Among families with membrane defects and complex intra-family phenotypes, we were able to identify a patient with mixed HS/HE who carried two variants in *SPTA1* and *SLC4A1* gene.

Notably, in addition to the disease-causing mutations, four patients also displayed heterozygous variants in other genes associated with hemolytic anemias (see Table 1). Although a synergistic effect of heterozygous variants has been hypothesized by some authors (van Zwieten et al., 2015; Pereira et al., 2016), in the presented cases the contribution of these variants to the phenotype remains to be determined.

A careful analysis of Group 2 put in light the limitations of laboratory work-up of CHAs. Only two among the undiagnosed families were found to have HS, suggesting that most of the HS patients can be correctly diagnosed during first and second level screening tools (Fermo et al., 2021). On the other hand, 10 out of the 19 families belonging to this group had an enzyme defect; in five of them enzymopathies were previously excluded because of normal enzyme activity, in the remaining cases the enzyme assays were not performed due to recent transfusions or unavailability of the appropriate sample. RBC enzyme defects are often detected by NGS in cases of unexplained anemia (Roy et al., 2016) or in patients misdiagnosed as CDAII (Shefer Averbuch et al., 2018; Russo et al., 2018), suggesting that this approach may greatly improve the diagnosis of these disorders.

In this series two PK deficient cases carrying known pathogenic variants (p.R486W, p.T384M, and p.V320M) repeatedly showed normal PK activity (14.7 and 18.8 IU/gHb, respectively, reference range 11.9–16.5), in absence of clear explanation (reticulocyte/WBC contamination excluded). Information on sensitivity of spectrophotometric enzyme assays is scanty, in particular for the rarer defects (Bianchi et al., 2019) but, as shown in the present cohort of patients, it will be possibly available in the next future through the systematic analysis of undiagnosed cases by NGS technologies.

Interestingly, we identified a triosephosphate isomerase deficiency in a 7 month child presenting at the time of the study only hemolytic anemia without neuromuscular manifestations. An early diagnosis allowed to inform and prepare the family and to offer bone marrow transplantation (BMT) as a therapeutic option before that neurological damage became evident (Conway et al., 2018); BMT was refused by the parents and the patient developed neurological signs at the age of 18 months. Early diagnosis in red cell disorders by NGS technologies opens interesting landscapes and perspectives in view of future therapeutic approaches for these diseases (Garcia-Gomez et al., 2016; Grace et al., 2019; Dessy-Rodriguez et al., 2020; Hrizo et al., 2021).

Among enzymopathies, we also identified 3 cases with undiagnosed G6PD deficiency, an heterozygous woman and two men with G6PD variants associated with chronic hemolysis, conditions in some cases underdiagnosed. A correct diagnosis enabled to give specific recommendation regarding contraindication for drugs associated with hemolytic anemia in G6PD -deficient patients (Luzzatto et al., 2020).

Differently from the *PIEZO1* mutated cases, all the *KCNN4* mutated patients belonged to group 2, confirming that molecular testing, and in particular NGS approaches, are the only diagnostic tools able to identify Gardos channelopathy, given the absence of a specific clinical or biological phenotype in this disorder; in fact, although ektacytometry, and some RBC and reticulocytes indices (Picard et al., 2019, 2021) may help to differentiate *PIEZO1* from *KCNN4* cases, no hematological parameter or specific laboratory test enable to differentiate Gardos channelopathy from other CHAs.

**FIGURE 3:**
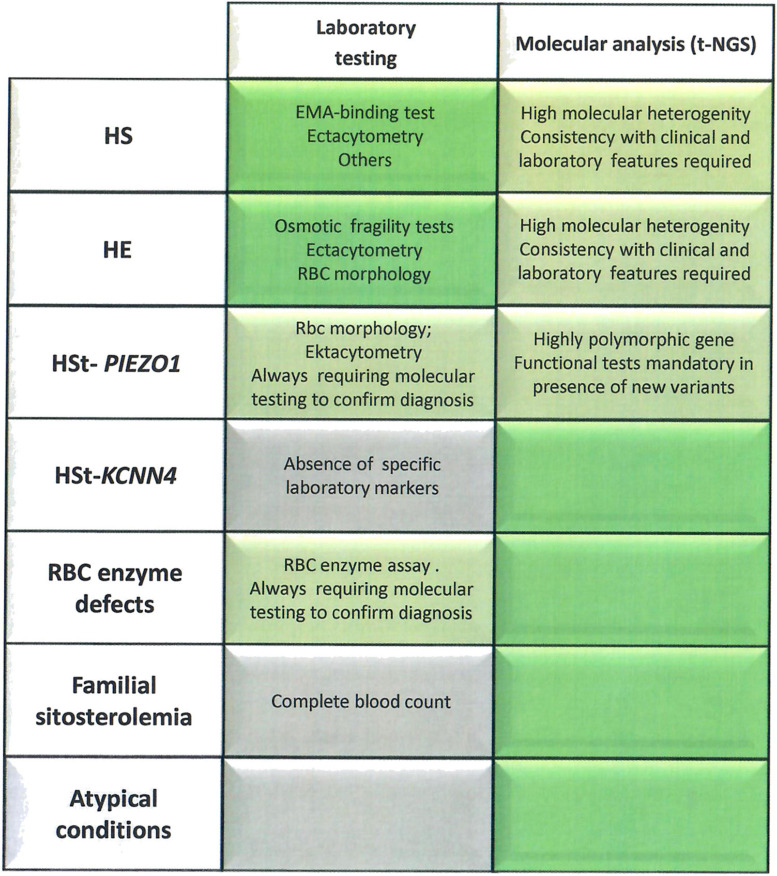
Advandages and limitations of laboratory approach vs. t-NGS technologies in congenital hemolytic anemias. The laboratory tests/parameters specifically useful for diagnosis of different diseases are reported. Brilliant green, exhaustive diagnostic approach; Green, requiring other approaches to confirm the diagnosis; gray, insufficient laboratory markers to reach the diagnosis.

In conclusion, by considering the entire cohort here studied, the implementation of diagnostic work-up with t-NGS analysis increased the number of diagnoses of patients with unexplained anemia up to 35%. As reported in Figure 3, cytoskeleton defects (HS/HE) are well diagnosed by laboratory approaches, deserving NGS only to atypical cases; on the other hand, the diagnosis of Gardos channelopathy, some RBC enzyme defects, familial siterosterolemia, X-linked defects in females and other rare and ultra-rare conditions definitely benefits of NGS approaches. However, it should be always considered that a set of functional tests is required to validate the molecular results and assess pathogenicity of the new identified variants, in particular in highly polymorphic genes such as *PIEZO1.*

## Data Availability Statement

The datasets presented in this study can be found in online repositories. The names of the repository/repositories and accession number(s) can be found in the article/Supplementary Table 1.

## Ethics Statement

The studies involving human participants were reviewed and approved by Fondazione IRCCS Ca’ Granda Ospedale Maggiore Policlinico. Written informed consent to participate in this study was provided by the participants’ legal guardian/next of kin.

## Author Contributions

EF and PB designed the study and performed NGS analysis and molecular studies. CV, AnZ, and AM performed laboratory investigations. WB, AZ, SP, EK, and JG followed-up patients. EF and PB drafted the manuscript, all other authors revised and approved the manuscript. All authors contributed to the article and approved the submitted version.

## Conflict of Interest

The authors declare that the research was conducted in the absence of any commercial or financial relationships that could be construed as a potential conflict of interest.

## Footnotes

1 http://wannovar.wglab.org/

2 http://sift.jcvi.org/

3 http://genetics.bwh.harvard.edu/pph2/

4 http://www.mutationtaster.org/

5 https://varsome.com/

6 www.hgvs.org/mutnomen

## Supplementary Material

Supplementary Table 1 Genes associated with CHAs investigated through a 43 genes-targeted Next Generation Sequencing panel.

## References

[B1] Agarwal A. M. Nussenzveig R. H. Reading N. S. Patel J. L. Sangle N. Salama M. E. (2016). Clinical utility of next-generation sequencing in the diagnosis of hereditary haemolytic anaemias. *Br. J. Haematol*. 174 806–814. 10.1111/bjh.14131 27292444

[B2] Berge K. E. Tian H. Graf G. A. Yu L. Grishin N. V. Schultz J. (2000). Accumulation of dietary cholesterol in sitosterolemia caused by mutations in adjacent ABC transporters. *Science* 290 1771–1775. 11099417 10.1126/science.290.5497.1771

[B3] Beutler E. Blume K. G. Kaplan J. C. Lohr G. W. Ramot B. Valentine W. N. (1977). International Committee for Standardization in Haematology: recommended methods for red-cell enzyme analysis. *Br. J. Haematol*. 35 331–340. 857853 10.1111/j.1365-2141.1977.tb00589.x

[B4] Bianchi P. Fermo E. Glader B. Kanno H. Agarwal A. Barcellini W. (2019). Addressing the diagnostic gaps in pyruvate kinase deficiency: Consensus recommendations on the diagnosis of pyruvate kinase deficiency. *Am. J. Hematol.* 94 149–161. 10.1002/ajh.25325 30358897 PMC7344868

[B5] Bianchi P. Fermo E. Vercellati C. Marcello A. P. Porretti L. Cortelezzi A. (2012). Diagnostic power of laboratory tests for hereditary spherocytosis: a comparison study in 150 patients grouped according to molecular and clinical characteristics. *Haematologica* 97 516–523. 10.3324/haematol.2011.052845 22058213 PMC3347664

[B6] Bianchi P. Vercellati C. Fermo E. (2020). How will next generation sequencing (NGS) improve the diagnosis of congenital hemolytic anemia?. *Ann. Transl. Med.* 8:268. 10.21037/atm.2020.02.151 32355712 PMC7186692

[B7] Choi H. O. Choi Q. Kim J. A. Im K. O. Park S. N. Park Y. (2019). Molecular diagnosis of hereditary spherocytosis by multi-gene target sequencing in Korea: matching with osmotic fragility test and presence of spherocyte. *Orphanet. J. Rare Dis*. 14:114. 10.1186/s13023-019-1070-0 31122244 PMC6533652

[B8] Chonat S. Risinger M. Sakthivel H. Niss O. Rothman J. A. Hsieh L. (2019). The Spectrum of SPTA1-Associated Hereditary Spherocytosis. *Front. Physiol.* 10:815. 10.3389/fphys.2019.00815 31333484 PMC6617536

[B9] Conway A. J. Brown F. C. Hortle E. J. Burgio G. Foote S. J. Morton C. J. (2018). Bone marrow transplantation corrects haemolytic anaemia in a novel ENU mutagenesis mouse model of TPI deficiency. *Dis. Model Mech.* 11:dmm034678. 10.1242/dmm.034678 29720471 PMC5992613

[B10] Del Orbe Barreto R. Arrizabalaga B. De la Hoz A. B. García-Orad Á Tejada M. I Garcia-Ruiz J. C. (2016). Detection of new pathogenic mutations in patients with congenital haemolytic anaemia using next-generation sequencing. *Int. J. Lab. Hematol*. 38 629–338. 10.1111/ijlh.12551 27427187

[B11] Dessy-Rodriguez M. Fañanas-Baquero S. A. Venturi V. Payán-Pernía S. Tornador C. Hernandez G. (2020). Quintana Bustamante, O. Modelling Congenital Dyserythropoietic Anemia Type II through Gene Editing in Hematopoietic Stem and Progenitor Cells. *Blood* 136 (Suppl. 1):27.

[B12] Fairbanks G. Steck T. L. Wallach D. F. H. (1971). Electrophoretic analysis of the major polypeptides of the human erythrocyte membrane. *Biochemistry* 10 2606–2617. 4326772 10.1021/bi00789a030

[B13] Fermo E. Monedero-Alonso D. Petkova-Kirova P. Makhro A. Pérès L. Bouyer G. (2020). Gardos channelopathy: functional analysis of a novel KCNN4 variant. *Blood Adv*. 4 6336–6341. 10.1182/bloodadvances.2020003285 33351129 PMC7756992

[B14] Fermo E. Vercellati C. Bianchi P. (2021). Screening tools for hereditary hemolytic anemia: new concepts and strategies. *Expert Rev. Hematol.* 1:11. 10.1080/17474086.2021.1886919 33543663

[B15] Fermo E. Vercellati C. Marcello A. P. Zaninoni A. Aytac S. Cetin M. (2019). Clinical and Molecular Spectrum of Glucose-6-Phosphate Isomerase Deficiency. Report of 12 New Cases. *Front. Physiol*. 10:467. 10.3389/fphys.2019.00467 31133865 PMC6514191

[B16] Fermo E. Vercellati C. Marcello A. P. Zaninoni A. van Wijk R. Mirra N. (2017). Hereditary Xerocytosis due to Mutations in PIEZO1 Gene Associated with Heterozygous Pyruvate Kinase Deficiency and Beta-Thalassemia Trait in Two Unrelated Families. *Case Rep. Hematol.* 2017:2769570. 10.1155/2017/2769570 28367341 PMC5358460

[B17] Garcia-Gomez M. Calabria A. Garcia-Bravo M. Benedicenti F. Kosinski P. López-Manzaneda S. (2016). Safe and Efficient Gene Therapy for Pyruvate Kinase Deficiency. *Mol. Ther.* 24 1187–1198. 10.1038/mt.2016.87 27138040 PMC5088764

[B18] Grace R. F. Rose C. Layton D. M. Galactéros F. Barcellini W. Morton D. H. (2019). Safety and Efficacy of Mitapivat in Pyruvate Kinase Deficiency. *N. Engl. J. Med.* 381 933–944. 10.1056/NEJMoa1902678 31483964

[B19] Hrizo S. L. Eicher S. L. Myers T. D. McGrath I. Wodrich A. P. K. Venkatesh H. (2021). Identification of protein quality control regulators using a Drosophila model of TPI deficiency. *Neurobiol. Dis.* 15:105299. 10.1016/j.nbd.2021.105299 33600953 PMC7993632

[B20] Kedar P. S. Gupta V. Dongerdiye R. Chiddarwar A. Warang P. P. Madkaikar M. R. (2019a). Molecular diagnosis of unexplained haemolytic anaemia using targeted next-generation sequencing panel revealed (p.Ala337Thr) novel mutation in GPI gene in two Indian patients. *J. Clin. Pathol*. 72 81–85. 10.1136/jclinpath-2018-205420 30337328

[B21] Kedar P. S. Harigae H. Ito E. Muramatsu H. Kojima S. Okuno Y. (2019b). Study of pathophysiology and molecular characterization of congenital anemia in India using targeted next-generation sequencing approach. *Int. J. Hematol.* 110 618–626. 10.1007/s12185-019-02716-9 31401766

[B22] King M. J. Garçon L. Hoyer J. D. Iolascon A. Picard V. Stewart G. (2015). International Council for Standardization in Haematology. ICSH guidelines for the laboratory diagnosis of nonimmune hereditary red cell membrane disorders. *Int. J. Lab. Hematol*. 37 304–325. 10.1111/ijlh.12335 25790109

[B23] Kopanos C. Tsiolkas V. Kouris A. Chapple C. E. Albarca Aguilera M. Meyer R. (2019). VarSome: the human genomic variant search engine. *Bioinformatics* 35 1978–1980. 10.1093/bioinformatics/bty897 30376034 PMC6546127

[B24] Laemmli U. K. (1970). Cleavage of structural proteins during the assembly of the head of bacteriophage T4. *Nature* 227 680–685. 5432063 10.1038/227680a0

[B25] Luzzatto L. Ally M. Notaro R. (2020). Glucose-6-phosphate dehydrogenase deficiency. *Blood* 136 1225–1240. 10.1182/blood.2019000944 32702756

[B26] Mansour-Hendili L. Aissat A. Badaoui B. Sakka M. Gameiro C. Ortonne V. (2020). Exome sequencing for diagnosis of congenital hemolytic anemia. *Orphanet. J. Rare Dis.* 15:180. 10.1186/s13023-020-01425-5 32641076 PMC7341591

[B27] Mariani M. Barcellini W. Vercellati C. Marcello A. P. Fermo E. Pedotti P. (2008). Clinical and hematologic features of 300 patients affected by hereditary spherocytosis grouped according to the type of the membrane protein defect. *Haematologica* 93 1310–1317. 18641031 10.3324/haematol.12546

[B28] Niss O. Chonat S. Dagaonkar N. Almansoori M. O. Kerr K. Rogers Z. R. (2016). Genotype-phenotype correlations in hereditary elliptocytosis and hereditary pyropoikilocytosis. *Blood Cells Mol. Dis.* 61 4–9. 10.1016/j.bcmd.2016.07.003 27667160 PMC5098801

[B29] Pereira J. Bento C. Maco L. Gonzalez A. Vagace J. Ribeiro M. L. (2016). Congenital dyserythropoietic anemia associated to a GATA1 mutation aggravated by pyruvate kinase deficiency. *Ann. Hematol.* 95 1551–1553. 10.1007/s00277-016-2720-0 27342114

[B30] Picard V. Guitton C. Mansour-Hendili L. Jondeau B. Bendélac L. Denguir M. (2021). Rapid Gardos Hereditary Xerocytosis Diagnosis in 8 Families Using Reticulocyte Indices. *Front. Physiol.* 11:602109. 10.3389/fphys.2020.602109 33519508 PMC7841495

[B31] Picard V. Guitton C. Thuret I. Rose C. Bendelac L. Ghazal K. (2019). Clinical and biological features in PIEZO1-hereditary xerocytosis and Gardos channelopathy: a retrospective series of 126 patients. *Haematologica* 104 1554–1564. 10.3324/haematol.2018.205328 30655378 PMC6669138

[B32] Richards S. Aziz N. Bale S. Bick D. Das S. Gastier-Foster J. (2015). ACMG Laboratory Quality Assurance Committee. Standards and guidelines for the interpretation of sequence variants: a joint consensus recommendation of the American College of Medical Genetics and Genomics and the Association for Molecular Pathology. *Genet. Med.* 17 405–424. 10.1038/gim.2015.30 25741868 PMC4544753

[B33] Roy N. B. A. Wilson E. A. Henderson S. Wray K. Babbs C. Okoli S. (2016). A novel 33-Gene targeted resequencing panel provides accurate, clinical-grade diagnosis and improves patient management for rare inherited anaemias. *Br. J. Haematol.* 175 318–330. 10.1111/bjh.14221 27432187 PMC5132128

[B34] Russo R. Andolfo I. Manna F. Gambale A. Marra R. Rosato B. E. (2018). Multi-gene panel testing improves diagnosis and management of patients with hereditary anemias. *Am. J. Hematol.* 93 672–682. 10.1002/ajh.25058 29396846

[B35] Russo R. Marra R. Rosato B. E. Iolascon A. Andolfo I. (2020). Genetics and Genomics Approaches for Diagnosis and Research Into Hereditary Anemias. *Front. Physiol*. 11:613559. 10.3389/fphys.2020.613559 33414725 PMC7783452

[B36] Shefer Averbuch N. Steinberg-Shemer O. Dgany O. Krasnov T. Noy-Lotan S. Yacobovich J. (2018). Targeted next generation sequencing for the diagnosis of patients with rare congenital anemias. *Eur. J. Haematol*. 101 297–304. 10.1111/ejh.13097 29786897

[B37] Steinberg-Shemer O. Tamary H. (2020). Impact of next-generation sequencing on the diagnosis and treatment of congenital anemias. *Mol. Diagn Ther*. 4 397–407. 10.1007/s40291-020-00478-3 32557003

[B38] Svidnicki M. C. C. M. Zanetta G. K. Congrains-Castillo A. Costa F. F. Saad S. T. O. (2020). Targeted next-generation sequencing identified novel mutations associated with hereditary anemias in Brazil. *Ann. Hematol*. 99 955–962. 10.1007/s00277-020-03986-8 32266426 PMC7241966

[B39] Tzavella E. Hatzimichael E. Kostara C. Bairaktari E. Elisaf M. Tsimihodimos V. (2017). Sitosterolemia: A multifaceted metabolic disorder with important clinical consequences. *J. Clin. Lipidol.* 11 1095–1100. 10.1016/j.jacl.2017.04.116 28545928

[B40] van Vuren A. van der Zwaag B. Huisjes R. Lak N. Bierings M. Gerritsen E. (2019). The complexity of genotype-phenotype correlations in hereditary spherocytosis: a cohort of 95 patients. *HemaSphere* 3:4. 10.1097/HS9.0000000000000276 PMC6745925 31723846

[B41] van Zwieten R. van Oirschot B. A. Veldthuis M. Dobbe J. G. Streekstra G. J. van Solinge W. W. (2015). Partial pyruvate kinase deficiency aggravates the phenotypic expression of band 3 deficiency in a family with hereditary spherocytosis. *Am. J. Hematol.* 90 E35–E39. 10.1002/ajh.23899 25388786

[B42] Vives-Corrons J. L. Krishnevskaya E. Rodriguez I. H. Ancochea A. (2021). Characterization of hereditary red blood cell membranopathies using combined targeted next-generation sequencing and osmotic gradient ektacytometry. *Int. J. Hematol.* 113 163–174. 10.1007/s12185-020-03010-9 33074480

[B43] Wang G. Cao L. Wang Z. Jiang M. Sun X. Bai X. (2012). Macrothrombocytopenia/stomatocytosis specially associated with phytosterolemia. *Clin. Appl. Thromb. Hemost.* 18 582–587. 22297561 10.1177/1076029611435090

[B44] Wang Z. Cao L. Su Y. Wang G. Wang R. Yu Z. (2014). Specific macrothrombocytopenia/hemolytic anemia associated with sitosterolemia. *Am. J. Hematol*. 89 320–324. 24166850 10.1002/ajh.23619

[B45] Xue J. He Q. Xie X. Su A. Cao S. (2019). Clinical utility of targeted gene enrichment and sequencing technique in the diagnosis of adult hereditary spherocytosis. *Ann. Transl. Med.* 7:527. 10.21037/atm.2019.09.163 PMC6861754 31807509

[B46] Zaninoni A. Fermo E. Vercellati C. Consonni D. Marcello A. P. (2018). Use of Laser Assisted Optical Rotational Cell Analyzer (LoRRca MaxSis) in the Diagnosis of RBC Membrane Disorders, Enzyme Defects, and Congenital Dyserythropoietic Anemias: A Monocentric Study on 202 Patients. *Front. Physiol.* 9:451. 10.3389/fphys.2018.00451 29755372 PMC5934481

[B47] Zheng J. Ma J. Wu R. H. Zhang X. Su Y. Zhang R. (2019). Unusual presentations of sitosterolemia limited to hematological abnormalities: A report of four cases presenting with stomatocytic anemia and thrombocytopenia with macrothrombocytes. *Am. J. Hematol*. 94 E124–E127. 30697800 10.1002/ajh.25427

