# Supplementary material for: Targeted Next Generation Sequencing and Diagnosis of Congenital Hemolytic Anemias: A Three Years Experience Monocentric Study
Source: Front Physiol. 2021 May 21;12:684569. doi: 10.3389/fphys.2021.684569 (PMC8176228; doi:10.3389/fphys.2021.684569)
Supplement: Supplementary Table 1 — Genes associated with CHAs investigated through a 43 genes-targeted Next Generation Sequencing panel. [file Data_Sheet_1.doc]

**Supplemental Methods**

*LoRRca Osmoscan*

Osmoscan was performed by means of Laser-assisted Optical Rotation Cell Analyzer (LoRRca MaxSis, Mechatronics, Hoorn, The Netherlands) on 250 μl of EDTA blood sample suspended in 5 mL of polyvinylpyrrolidone buffer (PVP, Mechatronics, Hoorn, The Netherlands), according to the manufacturer's instructions.

The following parameters are evaluated: the Omin-value corresponding to the osmolality at which the deformability reaches its minimum and representing the 50% of the RBCs hemolysis in conventional osmotic fragility assays; the elongation index (EI) max corresponding to the maximal deformability or elongation obtained near the isotonic osmolality and expression of the membrane surface; the Ohyper (the osmolality in the hypertonic region corresponding to 50% of the EImax) reflecting the mean cellular hydration status; the area under the curve (AUC), defined in the provided software as the AUC beginning from a starting point in the hypo-osmolar region and an ending point in the hyper-osmolar region (instrument settings 500 mOsm/kg). The patients were classified following the range values of EImax, Omin, Ohyper, AUC reported in each subset of CHAs by Zaninoni et al (2018).

Supplemental Table 1

Genes associated with CHAs investigated through a 43 genes-targeted Next Generation Sequencing panel.

| **Gene** | **Ref. Sequence** | **Gene** | **Ref. Sequence** | **Gene** | **Ref. Sequence** |
| --- | --- | --- | --- | --- | --- |
| **ABCB6** | NM_005689.3 | **ABCG5** | [NM_022436.2](http://www.ncbi.nlm.nih.gov/entrez/viewer.fcgi?val=NM_022436.2) | **ABCG8** | [NM_022437.2](http://www.ncbi.nlm.nih.gov/entrez/viewer.fcgi?val=NM_022437.2) |
| **AK1** | [NM_000476.2](http://www.ncbi.nlm.nih.gov/entrez/viewer.fcgi?val=NM_000476.2) | **ALAS2** | [NM_000032.5](http://www.ncbi.nlm.nih.gov/entrez/viewer.fcgi?val=NM_000032.4) | **ALDOA** | NM_000034.2 |
| **ANK1** | [NM_000037.3](http://www.ncbi.nlm.nih.gov/entrez/viewer.fcgi?val=NM_000037.3) | **C15ORF41** | NM_001130010.2 | **CDAN1** | [NM_138477.2](http://www.ncbi.nlm.nih.gov/entrez/viewer.fcgi?val=NM_138477.2) |
| **ENO1** | NM_001428.4 | **EPB41** | [NM_004437.3](http://www.ncbi.nlm.nih.gov/entrez/viewer.fcgi?val=NM_004437.3) | **EPB42** | NM_000119.2 |
| **G6PD** | [NM_001042351.1](http://www.ncbi.nlm.nih.gov/entrez/viewer.fcgi?val=NM_001042351.1) | **GATA1** | [NM_002049.3](http://www.ncbi.nlm.nih.gov/entrez/viewer.fcgi?val=NM_002049.3) | **GCLC** | [NM_001498.3](http://www.ncbi.nlm.nih.gov/entrez/viewer.fcgi?val=NM_001498.3) |
| **GCLM** | [NM_002061.2](http://www.ncbi.nlm.nih.gov/entrez/viewer.fcgi?val=NM_002061.2) | **GLRX5** | NM_016417.2 | **GPI** | NM_000175.3 |
| **GPX1** | NM_000581.2 | **GSR** | NM_000637.4 | **GSS** | [NM_000178.2](http://www.ncbi.nlm.nih.gov/entrez/viewer.fcgi?val=NM_000178.2) |
| **HK1** | NM_033496.2 | **KCNN4** | [NM_002250](http://www.ncbi.nlm.nih.gov/nuccore/NM_002250).2 | **KIF23** | NM_138555.3 |
| **KLF1** | NM_006563.4 | **NT5C3A** | NM_016489.12 | **PFKL** | NM_001002021 |
| **PFKM** | NM_000289.5 | **PGK1** | NM_000291.3 | **PGM1** | [NM_002633.2](http://www.ncbi.nlm.nih.gov/entrez/viewer.fcgi?val=NM_002633.2) |
| **PKLR** | NM_000298.5 | **PIEZO1** | NM_001142864.3 | **RHAG** | NM_000324.2 |
| **SBDS** | NM_016038.3 | **SEC23B** | NM_006363.4 | **SLC11A2** | NM_000617.2 |
| **SLC2A1** | [NM_006516.2](http://www.ncbi.nlm.nih.gov/entrez/viewer.fcgi?val=NM_006516.2) | **SLC4A1** | NM_000342.3 | **SLC25A38** | NM_017875.2 |
| **SPTA1** | NM_003126.3 | **SPTB** | NM_001355436.1 | **STOM** | [NM_004099](http://www.ncbi.nlm.nih.gov/nuccore/NM_004099) |
| **TPI1** | NM_000365.5 |  |  |  |  |
